# Occupational safety of janitors in Ethiopian University during COVID-19 pandemic: Results from observational study

**DOI:** 10.3389/fpubh.2022.895977

**Published:** 2022-07-29

**Authors:** Chala Daba, Mesfin Gebrehiwot, Lechisa Asefa, Hailu Lemma, Amanuel Atamo, Edosa Kebede, Asha Embrandiri, Sisay Abebe Debela

**Affiliations:** ^1^Department of Environmental Health, College of Medicine and Health Sciences, Wollo University, Dessie, Ethiopia; ^2^Department of Environmental Health Science, Institute of Health, Bule Hora University, Bule Hora, Ethiopia; ^3^Department of Medical Laboratory Science, College of Medicine and Health Sciences, Ambo University, Ambo, Ethiopia; ^4^Department of Public Health, College of Medicine and Health Sciences, Salale University, Fitche, Ethiopia

**Keywords:** COVID-19, occupational safety, janitor, Ethiopia, University

## Abstract

**Introduction:**

Janitors are at high risk of COVID-19 infection, as they are among the frontline workers for the prevention and control of COVID-19. Poor occupational safety practices could contribute to loss of lives of janitors and the general public. However, there are no detailed investigations on occupational safety practices of janitors involved in different settings, such as universities where there are crowds of people. In addition, although observation is recognized as a better tool to investigate occupational safety practices, previous studies mainly employed self-administered questionnaires and/or face-to-face interviews as data collection mechanisms. Therefore, this study aimed to assess occupational safety practices to prevent COVID-19 transmission and associated factors among Ethiopian University janitors using an observation tool and a self-administered questionnaire.

**Methods:**

An institutional-based cross-sectional study was conducted among 410 janitors of Bule Hora University (Ethiopia) from November to December, 2021. A multivariable logistic regression model was used to identify the independent factors associated with occupational safety practices.

**Results:**

Occupational safety practices for COVID-19 were good only among 53.9% of the janitors. Training on COVID-19 prevention measures (AOR = 2.62; 95% CI: 1.57–4.37), availability of policy and protocol in the work place (AOR = 5.46; 95% CI: 3.57–8.36), and availability of soap/bleach (AOR = 2.71; 95% CI: 1.64–4.46) were found to significantly increase the likelihood of occupational safety of the janitors.

**Conclusion:**

A significant proportion of the janitors had poor occupational safety practices. Therefore, an adequate supply of PPE and regular training and awareness creation on COVID-19 should be strengthened. Close follow-up and regular supervision of safety procedures should also be conducted as controlling strategies.

## Background

Coronavirus disease 2019 (COVID-19) is a respiratory disease caused by severe acute respiratory syndrome coronavirus 2 (SARS-CoV-2). It was first reported in Wuhan, Hubei, province (China) during late December 2019 (1). The virus is mainly transmitted *via* respiratory droplets (2). The disease is known to have various symptoms ranging from no clinical symptoms to dry cough, fever, fatigue, and a severe form of respiratory illness (3, 4). A study conducted in China showed that the majority (80%) of the infected people had mild symptoms (5). Even though all age groups of the community are at risk of being infected with the virus, the risk of mortality and morbidity is higher among elders and patients with chronic diseases, such as asthma, hypertension, cancer, and heart and lung diseases (5).

The death rate due to COVID-19 has been alarming through time all over the world. As of 1 March 2022, it had affected more than 437 million people and caused more than 5.9 million deaths globally (6). COVID-19 could infect 7 billion people and cause 40 million deaths globally if immediate interventions are not taken (7). Similarly, over 11.1 million confirmed cases and 248,812 deaths were reported in Africa on 30 February 2022 (8). Beyond morbidity and mortality, coronavirus has devastating effects on the economy of a country. According to the Economic Commission for Africa (ECA), COVID-19 could cause up to 2.6% of gross domestic product (GDP) decline in the African continent (9).

A systematic review and meta-analysis study showed that more than 152,888 confirmed cases and 1,413 deaths of healthcare workers, including janitors, were reported globally on 8 June 2020 (10). Studies conducted in Bangladesh (11) and six other Asian countries (12) indicated that 11 and 8% of janitors, were infected with COVID-19 at their workplace. Among other healthcare workers, janitors are at high risk of getting infected with COVID-19 (13), as they are less knowledgeable of the concepts of self-hygiene and other COVID-19-related information (14). In addition, as SARS CoV-2 can be excreted through feces, janitors could be easily exposed to COVID-19 because of the nature of their work (15, 16).

Poor occupational safety practice could contribute to loss of lives of janitors and the general public (17). However, there are no detailed investigations on occupational safety practices among janitors involved in different settings, such as universities where there are many residents and visitors. In addition, although observation is recognized as a better tool to assess occupational safety practices (18), previous studies mainly employed self-administered questionnaires and/or face-to-face interviews (19–21) as data collection mechanisms. As a consequence, it is challenging to adequately inform the public about the risks involved and precautions needed. Therefore, there is an urgent need to investigate the actual occupational safety practices of janitors during routine activities.

This study was, therefore, aimed at assessing the occupational safety practices to prevent COVID-19 transmission and associated factors among Bule Hora University (Ethiopia) janitors using an observation tool and a self-administered questionnaire. The findings of this study would be helpful to identify the gaps in the struggle to prevent the spread of the virus among janitors in universities and other similar settings. This could have a significant role in taking necessary actions to reduce COVID-19 transmission. This is particularly important for countries like Ethiopia, where a large number of cases are being reported (fifth in Africa) (8).

## Materials and methods

### Study area

Bule Hora University is located some 467 km away from the Ethiopian capital, Addis Ababa. The University was established in 2015/16. Currently, it has a total of 17,120 students (10,542 regular and 6,578 extension), 1,153 academic staff, and 3,239 administrative staff (464 janitors). The University is serving as an isolation center for patients with COVID-19 and janitors who have close contact with infected people and materials.

### Study design, period, and population

An institutional-based cross-sectional study was conducted from November to December 2021 in Bule Hora University. All janitors in Bule Hora University were the source population, and randomly selected janitors from the registration logbook were the study population.

### Inclusion and exclusion criteria

All janitors in Bule Hora University who were actively working during the period of data collection were considered to be included in this study, whereas janitors who were on annual leave (*n* = 2) and who had a severe illness (*n* = 1) were excluded.

### Sample size determination and sampling procedure

The sample size was calculated using a single population proportion formula. A 5% margin of error (d), 95% confidence level (alpha, α = 0.05), and 50% proportion of good occupational safety practices of janitors were assumed. Accordingly, the total sample size was calculated as (22):


$$\begin{array}{l} \text{n}={\left(Z_{\alpha /2}\right)}^{2}p\;\left(1-p\right)/d^{2} \end{array}$$

where n = required sample size, Z = critical value for normal distribution at 95% confidence level (Z value at α = 0.05, two tailed = 1.96), *P* = 50% proportion of good occupational safety practice, and d = 5% margin of error.

By considering a 10% non-response rate, the final sample size for this study was 422. A simple random sampling technique (lottery method) was used to select the study participants from the list of 461 janitors.

### Data collection and quality assurance

The required data on occupational safety practice was collected using an observational checklist prepared after reviewing the appropriate literature (Appendix 1). Before the actual observation, four observers and a supervisor (environmental health experts) were trained for 2 days about the objectives of the study, data collection tools, and ethical issues to ensure the quality of the data. Detailed training on COVID-19 prevention measures was also given. During the observational study, the data collectors directly observed the study participants while conducting their routine activities. During observation, the janitors were unaware of the research activity to minimize bias (Hawthorne effect). Some other days after the completion of observation, a face-to-face interview was conducted on the same janitors to complete other required information (Appendix 2). The questionnaire was prepared in English and translated into the local language (Afan Oromo). Prior to the interview, a pre-test was conducted in a Bule Hora hospital (*n* = 20), and an amendment was made. The internal consistency of the questionnaire was checked using Cronbach's alpha coefficient and was found to be 0.82. The completeness and consistency of the data were checked daily by the supervisor, and daily feedback was given to the data collectors throughout the study.

### Data management and analysis

The collected data were checked, coded, and entered into EpiData version 3.1 and exported to SPSS version 25.0 for data cleaning and analysis. In order to identify factors associated with occupational safety practice, first, a bivariable logistic regression analysis with *p* < 0.25 was performed to screen candidate variables. Then, a multivariable analysis was conducted to control possible confounders. Adjusted odds ratios (AOR) and their 95% CI were used to measure the association between dependent and independent variables. A significance level of *p* < 0.05 was used to decide the significance of statistical tests. Multi-collinearity among the independent variables was assessed using standard error. As the maximum standard error was 1.95, there was no multicollinearity. Model fitness was checked by the Hosmer-Lemeshow test (23) with a *p*-value of 0.697, which indicated that the model was fit. The model also explained 78.3% of the variance in occupational safety practice.

### Study variables

The outcome variable for this study was occupational safety practice (good or poor) and the independent variables were sociodemographic characteristics (sex, age, educational status, marital status, religion, and experience), availability of personal protective equipment (facemask, glove, sanitizer, hand-washing facility, soap/bleach, and dust bin), and administrative control (presence of COVID-19 policy and protocol, and training) (Appendix 2).

### Operational definitions

To identify the level of occupational safety practice (good/poor), responses from 13 working practice questions were computed (Appendix 1). The correct practice (answer) for each item was given a score of “1,” and the incorrect practice was given a score of “0.” The mean score was used as a cut-off point. Accordingly, a janitor who correctly practiced above the mean was considered as having good occupational safety practice and vice versa.

### Safety precaution

All COVID-19 precautionary measures were considered by the data collectors as per the WHO guidelines.

## Results

In this study, a total of 410 janitors participated, giving a response rate of 97.2%. Table 1 shows the sociodemographic characteristics of the study participants. More than half (219, 53.4%) of the study participants were within the age group of 26–35 years. Similarly, 233 (56.8%) were women and 229 (55.9%) were married. Out of the 410 janitors, 276 (67.3%) had 3 years or more work experience (Table 1).

**Table 1:** Sociodemographic characteristics of the Bule Hora University janitors in Southern Ethiopia from November to December 2021.

**Variable**	**Category**	**Frequency (%)** **(*****n*** = **410)**	**Occupational safety**	
			**Good**	**Poor**
Sex	Female	233 (56.8%)	126	107
	Male	177 (43.2%)	95	82
Age	15–25	136 (33.2%)	71	65
	26–35	219 (53.4%)	125	94
	36 and above	55 (13.4%)	25	30
Educational status	No formal education	127 (31%)	65	62
	Primary (up to grade 8)	157 (38.3%)	93	64
	Secondary and above (grade 9–12)	126 (30.7%)	63	63
Experience	<3 years	134 (32.7%)	82	52
	≥3 years	276 (67.3%)	139	137
Marital status	Single	148 (36.1%)	80	68
	Married	229 (55.9%)	126	103
	Widowed	33 (8%)	15	18
Religion	Protestant	203 (49.5%)	111	92
	Orthodox	173 (42.2%)	89	84
	Muslim	34 (8.3%)	21	13

### Availability of personal protective equipment and sources of information about COVID-19

About two-thirds (67.1%) of the janitors had been provided with facemasks and gloves to wear during their routine work. More than half (54.1%) of the janitors reported the availability of hand-washing facility near their working area. Similarly, half (50.5%) of the janitors were provided with dust bins to dispose off used personal protective equipment, including gloves and face masks (Table 2).

**Table 2:** Availability of personal protective equipment to prevent COVID-19 transmission among the janitors of the Bule Hora University, Southern Ethiopia from November to December 2021.

**Variable**	**Category**	**Frequency** **(%)**	**Occupational safety**	
			**Good**	**Poor**
Availability of face mask to wear while cleaning	Yes	275 (67.1%)	160	115
	No	135 (32.9%)	61	74
Availability of glove while cleaning	Yes	275 (67.1%)	160	115
	No	135 (32.9%)	61	74
Availability of sanitizers	Yes	234 (57.1%)	146	88
	No	176 (42.9%)	75	101
Presence of hand washing facility	Yes	222 (54.1%)	131	91
	No	188 (45.9%)	89	98
Availability of soap/bleach	Yes	218 (53.2%)	149	69
	No	192 (46.8%)	72	120
Availability of dust bin	Yes	207 (50.5%)	127	80
	No	203 (49.5%)	94	109
Information about COVID-19	Yes	214 (52.2%)	104	109
	No	196 (47.8%)	116	80
	Social media	177 (83%)	82	95
	Google	103 (48%)	68	35
	Government media	132 (62%)	84	48
Source of information (*n* = 214)	Family/friends	103 (48%)	56	47

Surprisingly, only 214 (52.2%) had detailed information about COVID-19. Of the respondents who had information, the sources for more than three-fourths (83.7%) of the participants were social media (particularly Facebook) followed by government media (61.7%) (Table 2).

### Administrative control factors

Table 3 shows the administrative control factors to prevent COVID-19 transmission. More than half (53.9%) of the respondents reported the presence of COVID-19 prevention policy and protocol in their working area. More than half (57.3%) of the janitors did not take any training on COVID-19 pandemic prevention.

**Table 3:** Administrative control factors to prevent COVID-19 transmission in the Bule Hora University, Southern Ethiopia from November to December 2021.

**Variable**	**Category**	**Frequency**	**Occupational safety**	
			**Good**	**Poor**
Presence of policy and protocol toward COVID-19 prevention	Yes	221 (53.9%)	160	61
	No	189 (46.1%)	61	128
Do you take training on COVID-19 prevention	Yes	175 (42.7%)	132	43
	No	235 (57.3%)	89	146

### Working practice of janitors toward COVID-19 prevention

Only 64.1 and 66.3% of the janitors had worn gloves and facemasks, respectively, while cleaning. Similarly, only 61 and 63.9% of them followed disinfection procedures and handled waste materials properly, respectively. Also, only 55.6% of the janitors avoided touching noses, faces, and eyes before hand-washing. In general, only 53.9% of the janitors had good occupational safety practice, while 46.1% had poor practice to prevent COVID-19 (Table 4).

**Table 4:** Safe working practice of the Bule Hora University janitors to prevent COVID-19 transmission from November to December 2021.

**Variables**	**Category**	
	**Frequency (%)**	**Frequency (%)**
	**Yes**	**No**
Wear glove while cleaning	263 (64.1%)	147 (35.9%)
Wear facemask while cleaning	272(66.3%)	138 (33.7%)
Wash hands before wearing PPE	269 (65.6%)	141 (34.4%)
Wash hands after removing PPE	284 (69.3%)	126 (30.7%)
Remove and discharge PPE after finishing surface cleaning	257 (62.7%)	153 (37.3%)
Change gloves after cleaning	273 (66.6%)	137 (33.4%)
Follow disinfection procedure	250 (61%)	160 (39%)
Handle waste material properly including face mask, glove and etc.	262 (63.9%)	148 (36.1%)
Wash hands after sneezing and coughing	258 (62.9%)	152 (37.1%
Avoid touching noses, faces and eyes before hand washing	228 (55.6%)	182 (44.4%)
Maintain social distancing	245 (59.8%)	165 (40.2%)
Wear protective shoes during cleaning	238 (58%)	172 (42%)
Wear gown during cleaning	206 (50.2%)	204 (49.8%)
Occupational safety of the janitors	Good	221(53.9%)
	Poor	189 (46.1%)

PPE, personal protective equipment.

### Factors associated with occupational safety practice to prevent COVID-19 transmission

In the multivariable logistic regression analysis, availability of soap, training on COVID-19, and presence of COVID-19 policy and protocol in the organization showed a significant association with safety practice of janitors toward COVID-19 prevention (Table 5).

**Table 5:** Multivariable analysis of factors associated with occupational safety practice among the janitors of Bule Hora University from November to December 2021.

**Variable**	**Category**	**Occupational safet**		**COR (95%CI)**	**AOR (95%CI)**	***p*** **-Value**
		**Good**	**Poor**			
Work experience	<3 years	82	52	1	1	0.071
	≥3 years	139	137	0.64 (0.420–0.972)	1.57 (0.96–2.56)	
Availability of face mask to wear while cleaning	Yes	160	115	1.67 (1.11–2.54	1.51 (0.91–2.49)	0.105
	No	61	74	1	1	
Availability of sanitizer	Yes	146	88	2.21 (1.48–3.13)	1.11 (0.66–1.85)	0.685
	No	75	101	1	1	
Presence of hand washing facility	Yes	131	91	1.58 (1.07–2.34)	0.81 (0.49–1.34)	0.420
	No	89	98	1	1	
Availability of soap/bleach	Yes	149	69	3.65 (2.42–5.49)	2.71 (1.64–4.46)	<0.001
	No	72	120	1	1	
Availability of dust bin	Yes	127	80	1.86 (1.25–2.75)	1.22 (0.74–2.01)	0.423
	No	94	109	1	1	
Information about COVID-19	Yes	104	109	1.52 (1.03–2.25)	1.2 (0.75–1.92)	0.445
	No	116	80	1	1	
Presence of COVID-19 policy and protocol	Yes	160	61	5.46 (3.57–8.36)	3.38 (2.07–5.49)	<0.001
	No	61	128	1	1	
Do you take training on COVID-19 prevention	Yes	132	43	5.09 (3.3–7.86)	2.62 (1.57–4.37)	<0.001
	No	89	146	1	1	

1, Reference category; COR, crude odds ratio; AOR, adjusted odds ratio; CI, confidence interval.

Workers who had access to soap or bleach in the working area were almost three times more likely to have good safety practice than those who had no soap or bleach in the working area (AOR = 2.71; 95% CI: 1.64–4.46). Similarly, janitors who had taken COVID-19 training were about three times more likely to have good practice than the others (AOR = 2.62; 95% CI: 1.57–4.37). The odds of good safety practice were 3.38 times higher among janitors who knew about the presence of COVID-19 policies and protocols than their counterparts (Table 5).

## Discussion

This study was conducted to determine the occupational safety practices of janitors and their associated factors at Bule Hora University (Ethiopia). In this study, only 53.9% of the janitors had good occupational safety practice to prevent COVID-19. This finding is consistent with previous study findings reported among healthcare professionals in Ethiopia (19). Our findings suggest that a significant portion of the janitors and, hence, clients and the general public are at risk of COVID-19 infection.

This study revealed that majority (67.1%) of the janitors had worn facemasks while they were cleaning. Indeed, this figure was lower than the report from a community study in Taiwan (98%) (24). In contrast, it was greater than that reported among the Gedeo community (Ethiopia) (25), Malaysian community (26), and healthcare providers in Bangladesh (20), which, respectively showed 6.3, 51, and 24.2% facemask-wearing. Similarly, in our study, two-thirds (67.1%) of the janitors had worn gloves while cleaning. This finding was lower than that found among healthcare workers in Bangladesh, which was reported to be 94.1%. The disparities could be attributed to differences in study period and supply/scarcity of personal protective equipment, such as facemasks and gloves.

In this study, three key risk factors (training on COVID-19 prevention measures, presence of COVID-19 policy and protocol, and availability of soap) were found to have a significant influence on the occupational safety practices of janitors. We found that janitors who attended training were more likely (about 3 times) to have good safety practice toward COVID-19 than the others. This suggests that one of the best ways for janitors to prevent COVID-19 infection is through regular training on COVID-19 prevention measures. This is due to the fact that trained and educated people are more likely to understand health education messages and implement COVID-19 prevention practices than non-trained and less-educated ones (27). Indeed, only 42.7% of the janitors attended training on COVID-19 prevention. Our results are consistent with a study finding reported among healthcare professionals in Bangladesh (26).

Similarly, janitors who knew the presence of COVID-19 policies and protocols in their working environment had 3.4 more chance of having good occupational safety practices than those who did not know about the policies and protocols. Unfortunately, only 53.9% of our study participants knew the presence of policies and procedures. This is similar to the findings in the Oromia region (Ethiopia) (19). Whenever there are detailed policies and procedures aimed at preventing COVID-19 in the workplaces, janitors will have the chance to read, know, and implement them. Consequently, they will have good practice. That is why the Ethiopian Ministry of Health had provided the protocol to universities.

Furthermore, the availability of soap/bleach in the working area was identified as an important factor related to occupational safety of janitors. In this study, janitors who had soap or bleach were 2.7 times more likely to have good safety practice toward COVID-19 than those who did not have soap in the working area. This is again concerning, as 46.8% of the janitors reported lack of soap/bleach. In the absence of soap/bleach, the hardworking and highly exposure-prone janitors will not have the opportunity to use them during their routine work activities.

## Conclusion

This study showed that only 53.9% of the janitors had good occupational safety practice toward COVID-19. The main factors that were found to significantly increase the likelihood of occupational safety practice of janitors were availability of soap, presence of COVID-19 policy and protocol, and training on COVID-19 prevention measures. Therefore, special attention should be given to supplying adequate PPE and regular training and awareness creation on COVID-19. Close follow-up and regular supervision of safety procedures should also be conducted as control strategies.

### Limitations of the study

Although the janitors were unaware of being observed, some might have suspected being under observation and thus modified their practice. In addition, due to shortage of literature on occupational safety practices of janitors toward COVID-19 prevention, the discussion was made on the basis of findings from different target groups and studies conducted in other areas. This cross-sectional study might not fully represent occupational safety practices throughout the year with varying workload periods and availability of resources overtime. Acknowledging these limitations, this observational study is suggested to provide a guide to future efforts in improving occupational safety of janitors in populated settings, such as universities.

## Data availability statement

The original contributions presented in the study are included in the article/Supplementary material, further inquiries can be directed to the corresponding authors.

## Ethics statement

The study was approved by the institutional review board of Bule Hora University, Institute of Health. Written informed consent for participation was not required for this study in accordance with the national legislation and the institutional requirements.

## Author contributions

CD, MG, LA, HL, and EK were involved in tool preparation, data analysis, interpretation, visualization, methods, and manuscript writing. AA, AE, and SAD were involved in data entry, collection, and editing. All authors contributed to the article and approved the submitted version.

## Conflict of interest

The authors declare that the research was conducted in the absence of any commercial or financial relationships that could be construed as a potential conflict of interest.

## Publisher's note

All claims expressed in this article are solely those of the authors and do not necessarily represent those of their affiliated organizations, or those of the publisher, the editors and the reviewers. Any product that may be evaluated in this article, or claim that may be made by its manufacturer, is not guaranteed or endorsed by the publisher.

## Abbreviations

AOR: adjusted odds ratio
CI: confidence interval
COR: crude odds ratio
COVID-19: coronavirus disease 2019
PPE: personal protective equipment.
