# Supplementary material for: Occupational safety of janitors in Ethiopian University during COVID-19 pandemic: Results from observational study
Source: Front Public Health. 2022 Jul 29;10:895977. doi: 10.3389/fpubh.2022.895977 (PMC9374277; doi:10.3389/fpubh.2022.895977)
Supplement: Supplementary file 1 [file Appendix_1.pdf]

# Occupational safety of janitors in Ethiopian University during COVID-19 pandemic: results from observational study

Chala Daba[1*+], Mesfin Gebrehiwot[1*+], Lechisa Asefa[2], Hailu Lemma[2], Amanuel Atamo[1], Edosa Kebede[3], Asha Embrandiri[1], and Sisay Abebe Debela[4]

*Correspondence: chaladaba293@gmail.com, **gebrehiwotmesfin@yahoo.com**

[1]Department of Environmental Health, College of Medicine and Health Sciences, Wollo University, Dessie, Ethiopia, P.O.B.1145

[2]Department of Environmental Health Science, Institute of Health, Bule Hora University, Bule Hora, Ethiopia, P.O.B. 144

[3]Departement of Medical Laboratory Science, College of Medicine and Health Sciences, Ambo University, Ambo, Ethiopia

[4]Department of Public Health, College of Medicine and Health Sciences, Salale University, Fitche, Ethiopia

†*These authors have contributed equally to this work*

**Appendix 1** Observational checklist used to collect data about the working practice of Bule Hora University janitors to prevent COVID-19 transmission, from November to December, 2021

Checklist ID __________

Name of the data collector ___________Signature_________Date ___/__/ 2021

Name of the supervisor ______________Signature __________Date ___/__/2021

| **S/no** | **Question** | **Response** | **Remark** |
|---|---|---|---|
| 1. | Wear glove while cleaning | 0=No<br>1= Yes | |
| 2. | Wear facemask while cleaning | 0=No<br>1= Yes | |
| 3. | Wash hands before wearing PPE | 0=No<br>1= Yes | |
| 4. | Wash hands after removing PPE | 0=No<br>1= Yes | |
| 5. | Remove and discharge PPE after finishing surface cleaning | 0=No<br>1= Yes | |
| 6. | Change gloves after cleaning | 0= No<br>1= Yes | |
| 7. | Follow disinfection procedure | 0=No<br>1= Yes | |
| 8. | Handle waste material properly including face mask, glove and etc. | 0= No<br>1= Yes | |
| 9. | Wash hands after sneezing and coughing | 0=No<br>1= Yes | |
| 10 | Avoid touching noses, faces and eyes | 0= No | |

| | | | |
|---|---|---|---|
| | before hand washing | 1= Yes | |
| 11 | Maintain social distancing | 0=No<br>1= Yes | |
| 12 | Wear protective shoes during cleaning | 0= No<br>1= Yes | |
| 13 | Wear gown during cleaning | 0=No<br>1=Yes | |

*PPE- Personal protective equipment
