# Supplementary material for: Occupational safety of janitors in Ethiopian University during COVID-19 pandemic: Results from observational study
Source: Front Public Health. 2022 Jul 29;10:895977. doi: 10.3389/fpubh.2022.895977 (PMC9374277; doi:10.3389/fpubh.2022.895977)
Supplement: Supplementary file 2 [file Appendix_2.pdf]

Occupational safety of janitors in Ethiopian University during COVID-19 pandemic: results from observational study

Chala Daba[1*+], Mesfin Gebrehiwot[1*+], Lechisa Asefa[2], Hailu Lemma[2], Amanuel Atamo[1], Edosa Kebede[3], Asha Embrandiri[1], and Sisay Abebe Debela[4]

*Correspondence: chaladaba293@gmail.com, gebrehiwotmesfin@yahoo.com

[1]Department of Environmental Health, College of Medicine and Health Sciences, Wollo University, Dessie, Ethiopia, P.O.B.1145

[2]Department of Environmental Health Science, Institute of Health, Bule Hora University, Bule Hora, Ethiopia, P.O.B. 144

[3]Departement of Medical Laboratory Science, College of Medicine and Health Sciences, Ambo University, Ambo, Ethiopia

[4]Department of Public Health, College of Medicine and Health Sciences, Salale University, Fitche, Ethiopia

†*These authors have contributed equally to this work*

Appendix 2 Questionnaire used to collect data about occupational safety practices towards COVID-19 and associated factors among Bule Hora University janitors, from November to December, 2021

Questionnaire ID __________

Name of the data collector ___________Signature_________Date ___/__/ 2021

Name of the supervisor ______________Signature __________Date ___/__/2021

**Part 1: Socio-demographic characteristics of Bule Hora University janitors, from November to December, 2021**			
**S/no**	**Question**	**Response**	**Remark**
1.	Age?	_________years	
2.	Sex?	1=Female<br>2= Male	
3.	Educational status?	1= No formal education<br>2= Primary (up to grade 8)<br>3= Secondary and above (grade 9-12)	
4.	Experience?	1= < 3 years<br>2= ≥ 3 years	
5.	Marital status?	1= Single<br>2= married<br>3= Widowed	
6.	Religion?	1= protestant<br>2= orthodox<br>3= Muslim	
**Part 2: Source of information about COVID-19 among of janitors of Bule Hora University, form November to December, 2021**			

7.	Source of information	1= Social media<br>2= Google<br>3= Government media<br>4= Family/ friend	**More than one answer is possible**
**Part 3: Availability of personal protective equipment to prevent COVID-19 transmission among janitors of Bule Hora University, from November to December, 2021**			
8.	Availability of face mask to wear while cleaning	0= No<br>1= Yes	Observe that moments
9.	Availability of glove while cleaning	0=No<br>1= Yes	Check by observation
10	Availability of sanitizers	0= No<br>1= Yes	Check by observation
11	Presence of hand washing facility	0=No<br>1=Yes	Check by observation
12	Availability of soap/bleach	0= No<br>1= Yes	Check by observation
13	Availability of dust bin	0= No<br>1=Yes	Check by observation
**Part 4: Administrative control characteristics of Bule Hora University Janitors to prevent COVID- 19 transmission, from November to December, 2021**			
14	Presence of policy and protocol towards COVID-19 prevention	0= No<br>1=Yes	
15	Training on COVID-19 prevention	0= No<br>1= Yes	
